# Intracellular Trafficking and Synaptic Function of APL-1 in *Caenorhabditis elegans*

**DOI:** 10.1371/journal.pone.0012790

**Published:** 2010-09-20

**Authors:** Mary Wiese, Adam Antebi, Hui Zheng

**Affiliations:** 1 Huffington Center on Aging and Department of Molecular and Human Genetics, Baylor College of Medicine, Houston, Texas, United States of America; 2 Max Planck Institute for Biology of Aging, Köln, Germany; New York State Institute for Basic Research, United States of America

## Abstract

**Background:**

Alzheimer's disease (AD) is a neurodegenerative disorder primarily characterized by the deposition of β-amyloid plaques in the brain. Plaques are composed of the amyloid-β peptide derived from cleavage of the amyloid precursor protein (APP). Mutations in APP lead to the development of Familial Alzheimer's Disease (FAD), however, the normal function of this protein has proven elusive. The organism *Caenorhabditis elegans* is an attractive model as the amyloid precursor-like protein (APL-1) is the single ortholog of APP, and loss of *apl-1* leads to a severe molting defect and early larval lethality.

**Methodology/Principal Findings:**

We report here that lethality and molting can be rescued by full length APL-1, C-terminal mutations as well as a C-terminal truncation, suggesting that the extracellular region of the protein is essential for viability. RNAi knock-down of *apl-1* followed by drug testing on the acetylcholinesterase inhibitor aldicarb showed that loss of *apl-1* leads to aldicarb hypersensitivity, indicating a defect in synaptic function. The aldicarb hypersensitivity can be rescued by full length APL-1 in a dose dependent fashion. At the cellular level, kinesins UNC-104/KIF-1A and UNC-116/kinesin-1 are positive regulators of APL-1 expression in the neurons. Knock-down of the small GTPase *rab-5* also leads to a dramatic decrease in the amount of *apl-1* expression in neurons, suggesting that trafficking from the plasma membrane to the early endosome is important for *apl-1* function. Loss of function of a different small GTPase, UNC-108, on the contrary, leads to the retention of APL-1 in the cell body.

**Conclusions/Significance:**

Our results reveal novel insights into the intracellular trafficking of APL-1 and we report a functional role for APL-1 in synaptic transmission.

## Introduction

Alzheimer's disease (AD) is a progressive neurodegenerative disorder characterized by the deposition of β-amyloid plaques, loss of cholinergic neurons and accumulation of neurofibrillary tangles within the brain. Plaques are primarily composed of the amyloid-β peptide derived from cleavage of the amyloid precursor protein (APP). Despite the discovery of dominant mutations in APP that lead to the development of Familial Alzheimer's Disease, the normal functional role of this protein within the neuron is still unclear. Past studies have implicated APP in cell adhesion, synaptogenesis, cell migration, signaling, apoptosis, axonal transport as well as development of the neuromuscular junction suggesting that APP is not restricted to a single function (See [1] for review).

APP is a type I trans-membrane protein that is conserved from *C. elegans* to humans. APP knock-out mice are viable and fertile and have mild defects in locomotor activity, forelimb grip strength, behavior and long term potentiation (LTP) [2], [3], [4], [5]. The subtlety of these phenotypes is thought to be due to functional redundancy with the two other members of the APP family, APLP1 and APLP2 as loss of APLP2 along with one of the other two APP homologs results in early postnatal lethality in mice [6], [7]. Because of the redundancy of these homologs, using the mammalian system to study the function of APP has proven challenging.

The *C. elegans* model offers a simplification of the mammalian system in that APL-1 is the only APP ortholog in the nematode and a null mutation leads to early larval lethality [8]. APL-1 is structurally similar to its mammalian counterpart and shares three major regions of homology: the N-terminal E1 and E2 domains and the highly conserved intracellular C-terminal domain [9]. APL-1 does not contain the amyloid-β sequence, similar to the functionally redundant mammalian APP homologs APLP1 and APLP2 [10], [11], [12].

In this study, we use *C. elegans* to investigate the normal functional role of APL-1. We report that APL-1 is necessary for viability, molting and regulation of neurotransmission. Full length rescue of the synaptic transmission defect is dose dependent, while the N-terminus of APL-1 is sufficient to rescue the molting and lethality phenotypes. At the cellular level, proper localization and protein levels of APL-1 throughout the neuron are dependent on the kinesin transporters UNC-104/KIF1A and UNC-116/kinesin-1 as well as the small GTPase RAB-5 and UNC-108/Rab2, indicating their likely role in APL-1 vesicle transport and endocytosis.

## Materials and Methods

### *C. elegans Strains*

Strains were cultivated at 20° as described previously [13]. The strains used in this study include the wild-type Bristol N2 strain, *apl-1(tm385)/lon-2(e678)*, *lon-2(e678)*, *dgk-1(nu62)*, *unc-32(e189)*, *unc-49(e372)*, *rrf-3(pk1426)*, *unc-104(e1265)*, *unc-116(e2310)*, *unc-108(n3263)*. Crosses were performed using standard genetics methods and final strains identified by phenotype and PCR.

### RNAi

RNAi was performed by feeding as previously described [14]. RNAi clones were isolated from the Ahringer RNAi library (Gene Service) by streaking clones onto plates containing 10 µg/ml tetracycline and 100 µg/ml ampicillin. Before use, all RNAi clones isolated from the library were validated by sequencing. Cultures were grown overnight in LB containing 100 µg/ml ampicillin and used to seed NGM plates containing 1 mM IPTG and 50 µg/ml ampicillin.

### Development and Brood Size Assays

Ten to twelve young adult worms were placed on individual RNAi plates or NGM plates and allowed to lay eggs overnight. The eggs were collected onto new plates and counted. After 48 hours the worms were scored for their developmental stage. For brood size assays, ten L4 worms were placed on individual NGM plates. Every 24 hours the number of eggs the worms produced were counted and the mothers transferred to new plates until no more eggs were produced. Brood size assays were analyzed by one-way ANOVA with Bonferroni post-hoc test.

### Movement Assays

Body bends per minute were obtained by placing late L4 worms individually on NGM plates and screening 24 hrs later. Body bends were counted over a 3 min time period and then divided to calculate the average number of body bends in one minute. One body bend is completed when the point behind the pharynx reaches the opposite apex of the sinusoidal curve. Measurements were statistically analyzed by one-way ANOVA with Bonferroni post-hoc test.

### Plasmid Construction

All constructs were generated, unless otherwise described, by amplifying target sequences with Phusion High-Fidelity DNA Polymerase (Finnzymes) using primers with overhanging restriction sites. The PCR products were digested with their respective restriction enzymes and ligated to the destination vector backbone. For *apl-1* expression studies and rescue experiments, worm *apl-1* genomic coding DNA was amplified from cosmid C42D8 (Sanger Institute) including 4.4 kb of sequence upstream from the start codon. This fragment was ligated into L3781 (Addgene plasmid 1590; Fire Lab *C. elegans* Vector Kit, 1999 plate) in frame with the GFP sequence at the C-terminal end of *apl-1* to generate *papl-1::apl-1::gfp*. The C-terminal truncation construct *papl-1::apl-1ΔIC::gfp* was generated by amplifying the coding region of *apl-1* excluding the sequence for the last 36 amino acids. This PCR product was cloned into L3781 followed by cloning the *apl-1* promoter upstream. Constructs containing *papl-1::apl-1ΔYENPTY::gfp*, *papl-1::apl-1::T658A::gfp*, and *papl-1::T658E::gfp* were generated from the original *papl-1::apl-1::gfp* full length construct using site-directed mutagenesis to introduce the individual mutations. For human rescue studies, human APP cDNA containing an mRFP fusion was amplified from N1-APP-RFP and cloned into the L3781 expression vector that had been cut with XmaI and NheI to remove the coding region of GFP. The *apl-1* promoter was amplified from C42D8 then added to the APP::RFP clone to generate *papl-1*::APP::RFP. APLP1 and APLP2 cDNA sequences were amplified from clones 3865417 and 2820109 (Open Biosystems) respectively and also cloned into the L3781 vector with the GFP tag on the 3′ end of the gene followed by the insertion of the *apl-1* promoter.

Plasmids for colocalization purposes were made by amplifying mCherry from the vector pCFJ90 (Addgene plasmid 19327; [15]) and cloning the sequence upstream of the start codon of either *rab-5* or *unc-108* cDNA. For *rab-5*, the mCherry sequence was cloned into pBZ103 containing *phsp16/2::rab-5* followed by cloning the *apl-1* promoter to generate the final construct containing *papl-1::*mCherry*::rab-5*. The *unc-108* construct was developed by inserting the amplified mCherry sequence into pAOLO174 containing *punc-108::unc-108*(Q65L). The Q65L mutation was removed using site-directed mutagenesis to generate the WT sequence. pBZ103 and pAOLO174 were generous donations from the Zhou lab at Baylor College of Medicine.

All constructs were completely sequenced to verify accuracy of promoters and coding regions. All primers used for cloning are referenced in File S1.

### Transgenic Strains

Transgenic strains were generated through microinjection of DNA constructs into the worm gonad as previously described [16]. For expression studies, *papl-1::apl-1::gfp* (40 ng/µl) was co-injected with the marker construct pRF4 (75 ng/µl), which contains *rol-6(su1006)*. The rescue constructs described above were individually injected into the gonads of the *apl-1(tm385)/lon-2(e678)* strain. F1 progeny containing the array were individually cloned onto new plates and the F2 progeny of the lines were analyzed to determine if rescue of the L1 lethality occurred by absence of both long worms, indicating loss of the balancer, and arrested L1 progeny. Presence of the *tm385* deletion was tested by PCR. The full length *apl-1* 10 ng/µl expressing strain was integrated by UV irradiation, outcrossed 3X with the *lon-2(e678)* strain to maintain the *tm385* deletion then crossed to the *rrf-3(pk1426)* background for RNAi studies. For colocalization experiments, constructs were co-injected into N2 worms. Rescue strain construct concentrations are listed in Table 1 with each being co-injected with the *pmyo-3*::CFP marker (L4816; Fire Lab *C. elegans* Vector Kit, 1999 plate) (20 ng/µl) and pCR2.1 added to a final injection concentration of 100 ng/µl. For colocalization experiments the following injection concentrations were used: *papl-1::apl-1::gfp* (20 ng/µl), *papl-1::*mCherry*::rab-5* (20 ng/µl), *punc-108::*mCherry*::unc-108* (5 ng/µl); pRF4 (75 ng/µl). Strains created in this study are listed in File S1. Representative strains are listed where multiple strains were generated at the same concentration.

**Table 1:** Summary of injection constructs and their rescue of *apl-1(tm385)* lethality.

Constructs^a^	Concentration	Rescue/Total Strains
*papl-1* b *::apl-1::gfp*	20 ng/µl	4/4
	10 ng/µl	4/4
*papl-1::apl-1*	20 ng/µl	3/3
	10 ng/µl	3/3
	5 ng/µl	3/3
*papl-1::apl-1*Δ*IC::gfp*	20 ng/µl	0/3
	10 ng/µl	8/8
*papl-1::apl-1* T658A*::gfp*	20 ng/µl	8/8
*papl-1::apl-1* T658E*::gfp*	20 ng/µl	3/3
*papl-1::apl-1*Δ*YENPTY::gfp*	20 ng/µl	4/4
	10 ng/µl	7/7
*papl-1::*APP*::*RFP	40 ng/µl	0/3
	20 ng/µl	0/2
	10 ng/µl	0/3
*papl-1::*APLP1*::gfp*	20 ng/µl	0/2
	10 ng/µl	0/3
*papl-1::*APLP2*::gfp*	20 ng/µl	0/2
	10 ng/µl	0/2
*papl-1::*APP*::*RFP*; papl-1::*APLP1*::gfp; papl-1::*APLP2*::gfp*	20 ng/µl each	0/3

a Constructs were used to inject *apl-1(tm385)/lon-2(e678)* and the F2 progeny were examined for the generation of a rescue strain.

b Endogenous *apl-1* promoter.

### Aldicarb Assay

L4 hermaphrodites were placed on control (L4440) or *apl-1* RNAi plates and allowed to mature and lay eggs overnight. Young adults from the F1 progeny were placed onto NGM plates containing 1 mM aldicarb (PS734, Sigma-Aldrich) in the presence of food then scored semi-blind for response to a harsh touch every 10 minutes for 2 hours. Animals unable to respond to the touch were scored as paralyzed. Adult RNAi aldicarb experiments were performed 48 hrs after young adult animals were placed on the control (L4440) or *apl-1* RNAi plates. All rescue and mutant strains were tested as young adults. Strains were compared statistically by one-way ANOVA with Bonferroni post-hoc test.

### Protein Extraction and Western Blotting

Worms were synchronized by bleaching as previously described [17] and three 10 cm plates containing L4 stage worms were harvested. Samples were washed with TE, pelleted by centrifugation then placed at −80°C for at least 15 min. The pellet was thawed and all liquid replaced with 50 µl RIPA buffer containing protease inhibitors (Roche). Samples were sonicated twice for 10 seconds each then centrifuged at 10,500×g for 10 min at 4°C. Supernatants were collected and protein concentrations were measured by plate reader using a detergent-compatible colorimetric protein assay (Bio-Rad). Samples were then combined with 2X loading buffer and boiled at 80°C for 10 min prior to loading. SDS-PAGE was performed by loading 20 µg of protein sample into a 5% SDS-polyacrylamide gel. Samples were transferred onto nitrocellulose membrane and then the membrane was blocked for 2 hrs in 5% milk diluted with PBST. Membranes were probed with antibodies against GFP 1∶5000 (ab290; Abcam) or *C. elegans* γ-tubulin 1∶1000 (ab50721;Abcam) overnight in blocking solution. Following incubation with primary antibodies, membranes were washed 3X for 10 min in PBST, incubated with 2° anti-Rabbit HRP-conjugated antibody 1∶5000 (Vector Laboratories) for 1 hr then washed again 3X for 10 min in PBST. Bands were detected using the Amersham ECL chemiluminescence reagent (GE Healthcare Life Sciences) and band density quantified using ImageJ software (National Institutes of Health). Band density was normalized to the loading control and compared using the Student's t-test.

### Fluorescence Microscopy and Quantification

Imaging was performed by placing live animals anesthetized with 20 mM sodium azide on a 2% agarose pad. Images were obtained using a Zeiss Axioscope 2 Plus upright microscope equipped with an Axiocam MRm camera and Axiovision 4.1 software. Pictures were acquired with 100X or 63X lens. In the case of fluorescence quantification, head neurons of 10–20 worms per genotype were imaged with identical exposure times. Each neuronal cell body was imaged at its widest diameter in the plane of focus. Fluorescence was quantified using ImageJ. Control pictures were taken on the same day to account for differences in bulb intensity. Genotypes were compared using the Student's t-test. Colocalization experiments were performed by taking images on an Olympus IX50 inverted microscope. Images underwent de-convolution using Metamorph software and then overlayed using ImageJ. Colocalization quantification was performed as previously described [18] using intensity correlation analysis with the following modifications. The neuronal cell body was set as the region of interest and then the intensity correlation quotient (ICQ) was quantified using ImageJ as described by the McMaster Biophotonics Facility. In brief, an ICQ of 0 indicates random staining between the two fluorescent images while 0<ICQ≤0.5 indicates colocalization and 0≥ICQ>−0.5 occurs during segregated staining. For further description of the analysis refer to Li et al. (2004) [18]. ICQ values of the neurons were statistically analyzed using the one-sample t-test to compare the mean ICQ values against a random staining value of 0.

### RNA Extraction and qRT-PCR

Worms were synchronized by bleaching and harvested at the L4 stage (∼6,000 per sample) by rinsing worms off the plate using TE. Samples underwent a freeze/thaw cycle 4X between liquid nitrogen and a 37°C waterbath followed RNA isolation using the Qiagen RNeasy Lipid Tissue kit method with the addition of the RNase free DNase steps (Qiagen). cDNA was generated by reverse transcription using the Superscript III First Strand kit (Invitrogen) with the input of equal concentrations (1000 ng/µl) of RNA as measured by NanoDrop (NanoDrop Technologies). PCR primers to test *apl-1* expression were designed using Primer Express 2.0 (Applied Biosystems) (apl-1 Fwd ACTCACAGTGTCAGACCGTACCA, apl-1 Rev GTGCGGGACTTGAAGAGCTT) and *ama-1* was used as the endogenous control (ama-1 qPCR f1 CACGCGTTCAGTTTGGAATTC, ama-1 qPCR r1, AACTCGACATGAGCCACTGACA). Dilutions of the cDNA samples were mixed with SYBR Green following the Power SYBR Green PCR Master Mix protocol (Applied Biosystems). Quantitative real-time PCR (qRT-PCR) was performed using the ABIprism 7000 and data collected using the 7000 System SDS Software (Applied Biosystems). Primer efficiencies were originally validated using the standard curve method with all subsequent experiments using a 1∶50 dilution of cDNA and results analyzed using the comparative C_t_ method. Bars represent the mean of a triplicate containing a single biological sample with error bars calculated from the sample and endogenous control standard deviations (STD  =  √((Sample STD)∧2+(Housekeeping Gene STD)∧2)).

## Results

### Characterization of *apl-1* Expression

**Figure 1:**
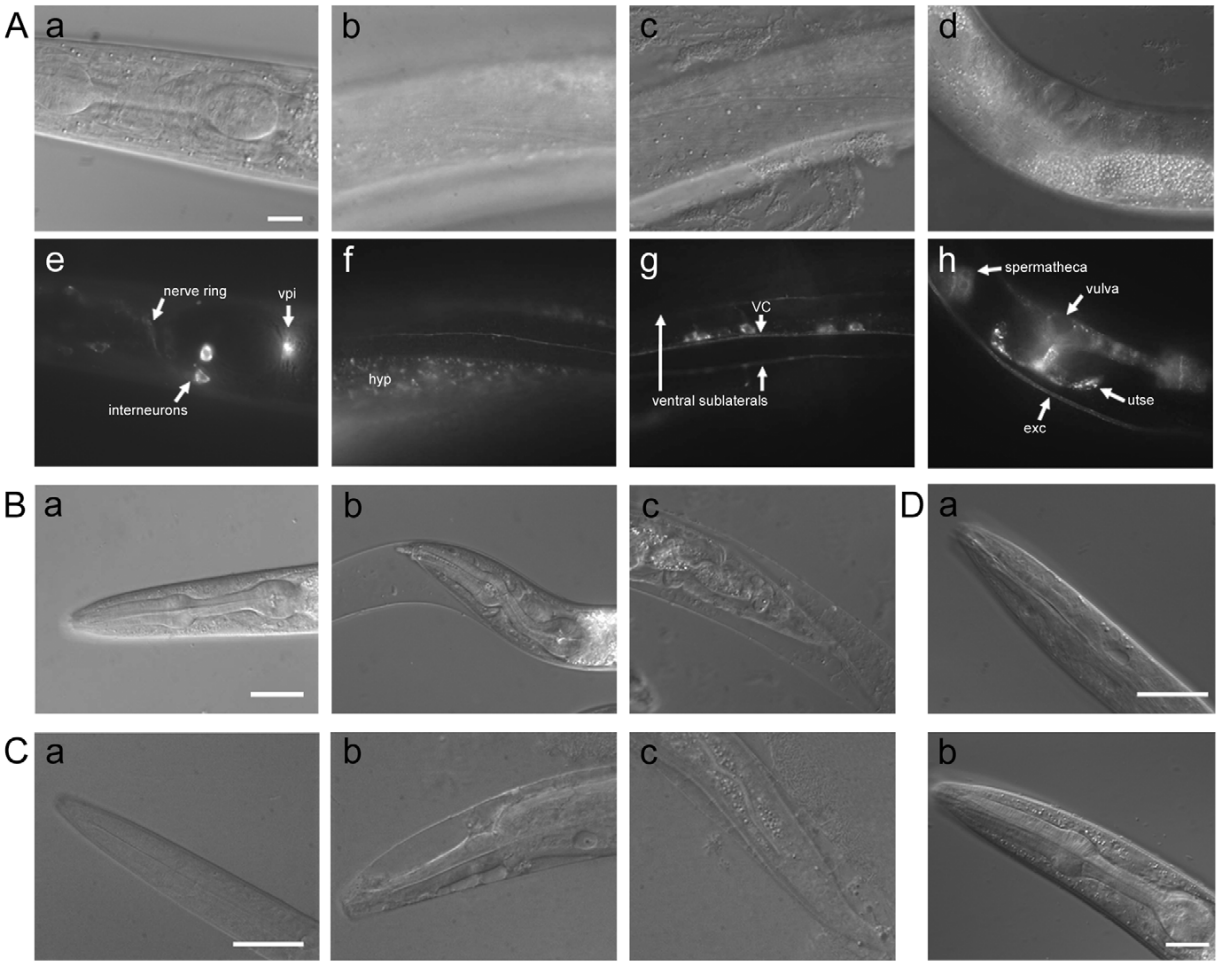
Characterization of *apl-1* expression and loss of function. (A(a–d)) DIC images corresponding with APL-1::GFP fluorescence (A(e–h)) respectively. vpi – pharyngeal intestinal valve; VC – ventral cord; exc- excretory cell; utse – uterine seam junction (Scale bar, 10 µm) (B) N2 L4 worm on control RNAi bacteria (L4440) (a), L4 worm with partial gene knock-down using *apl-1* RNAi showing loose cuticle in the head (b) and tail (c) region. (Scale bars, 20 µm) (C) N2 worm at L1 (a) compared to an *apl-1(tm385)* homozygous L1 arrest demonstrating degradation in the head (b) and tail (c). (Scale bar, 10 µm) (D) Rescue of the molting phenotype of the *apl-1* homozygous null worms by *apl-1::gfp* at L1 (a) and L4 stages (b). (Scale bars 10 µm and 20 µm respectively)

To determine the expression pattern of *apl-1*, transgenic worms were generated expressing an *apl-1::gfp* fusion protein driven by the endogenous *apl-1* promoter. APL-1::GFP fluorescence is detected in the cell bodies and processes of nerve ring interneurons (Figure 1 A,e) and the ventral cord (Figure 1 A,g). *apl-1::gfp* is also expressed in socket cells and amphids present in the head. Strong expression is seen in junctional cells such as the pharyngeal intestinal valve (Figure 1 A,e), which tethers the pharynx to the intestine, and the uterine seam junction in adults (Figure 1 A,h), which provides the structural connection between the epidermis and the uterus. APL-1 can be weakly detected in many epidermal epithelia including hyp7 (Figure 1 A,f), the hypodermal syncitium surrounding the worm, as well as vulval cells, rectal valve cells, pharyngeal arcade cells, and tail hypodermis. Expression is prominent in the excretory cell (Figure 1 A,h), a long H-shaped cell implicated in fluid balance. APL-1 was notably absent from body wall muscle and intestine. These expression patterns indicate that *apl-1* is active in cells with high levels of structural components such as synapses, junctional epithelial cells and cells with apical basal polarity.

### *apl-1* Loss of Function and Genetic Rescue

Similar to the molting defect caused by *apl-1* null mutations, knock-down of *apl-1* using RNAi on the RNAi sensitive *rrf-3(pk1426)* strain led to defective molting starting at the L3/L4 molt and continuing in the L4/YA molt (Figure 1B). A variety of molting phenotypes were seen which ranged from loose cuticle around the head and tail (7.5%, n = 173), internal pinching of the worm body at or just posterior to the head (4.6%), degradation around the mouth area (9.8%) or a cuticle plug around the mouth (38.1%) (Figure S1A, B). All of the worms had a very transparent appearance that, when examined at higher magnification, appeared as empty spaces in the worm spanning the length of the body. *apl-1* knock-down also led to delayed development, as most of the population after two days was in the L4 stage while the majority of the control worms had completed development to adulthood (Figure S1C). Furthermore, we observed that worms on *apl-1* RNAi exhibited sluggish movement, failing to move normally even when touched.

The *apl-1(tm385)* allele is a deletion that removes 646 base pairs including exon 3, which deletes 42 amino acids leading to a frame shift of the downstream sequence, resulting in a premature stop codon (Figure S2A). Worms homozygous for the *apl-1*(*tm385*) deletion are L1 lethal and exhibit internal vacuolization, degradation and loose cuticle phenotypes (Figure 1C) similar to previously reported null mutations [8], indicating that the *tm385* lesion is also null. We attempted to rescue the lethality of this mutant using constructs containing either full length *apl-1*, mutations within the highly conserved C-terminal domain, a C-terminal truncation of *apl-1*, or human *APP*, *APLP1* or *APLP2* (summarized in Table 1). All constructs were driven by the *apl-1* promoter and fused to a C-terminal GFP. Rescue constructs included a deletion of the highly conserved YENPTY motif which is known to bind to many different adaptor proteins or mutations of the conserved Thr668 residue (Thr658 in APL-1) which is a phosphorylation site that can regulate the localization and binding partners of APP [19], [20], [21], [22], [23]. The Thr658 site was mutated either to Ala (T658A) or Glu (T658E) to mimic the dephosphorylated and constitutively phosphorylated protein respectively. In addition, we created a C-terminal truncation construct (ΔIC) by removing the last 36 amino acids of the protein leaving the trans-membrane sequences intact rather than expressing only the soluble ectodomain of APL-1, attempting to maintain proper membrane anchoring and correct processing of the protein. To avoid any potential complications associated with the C-terminal GFP fusion used to monitor APL-1 expression, we also performed rescue experiments in parallel using full-length *apl-1* lacking the GFP tag.

Previous genetic rescue studies showed that the full length APL-1, the N-terminal sequences of APL-1, as well as the non-overlapping E1 or E2 fragments of APL-1 were sufficient to rescue *apl-1* null lethality [8]. These strains were generated by injecting very high concentrations of the DNA constructs (50–100 ng/µl), which consequently led to strong over-expression of *apl-1* and functional impairments including delayed development, sluggish movement and smaller brood sizes. This ectopic over-expression may bypass any intracellular trafficking or processing requirement needed for rescue under physiological conditions. We attempted to limit these potential over-expression side effects by injecting much lower concentrations of the expression vectors (ranging from 10–20 ng/µl). We found that the most highly expressing array on the N2 background displayed normal movement and brood sizes with only a slight developmental delay (Figure S2), indicating that the effect of over-expression is minimal at the DNA concentrations we used for injection.

When expressed on the *apl-1(tm385)* background, we found that full length *apl-1* along with all of the C-terminal mutation constructs were able to rescue the lethality and molting defects caused by the *tm385* deletion (Figure 1D, Table 1). Interestingly, while injection of *apl-1* ΔIC at 10 ng/µl rescued the lethality and molting, the construct was unable to rescue at the higher concentration of 20 ng/µl. The reason for this dose-dependent rescue is not clear as it is not seen in any of the other constructs.

In order to determine if human APP or one of its mammalian homologs APLP1 or APLP2 could act as functional homologs to *apl-1*, each of these genes expressed by the *apl-1* promoter were injected individually into *apl-1(tm385)* heterozygous worms to test for rescue of the *apl-1* lethality. Similar to the previous report of APP being unable to rescue *apl-1* null lethality [8], none of the human genes were able to rescue the *tm385* lethality, either expressed separately or together (Table 1).

### *apl-1* Deficiency Leads to Aldicarb Hypersensitivity Independent of the Molting Phenotype

**Figure 2:**
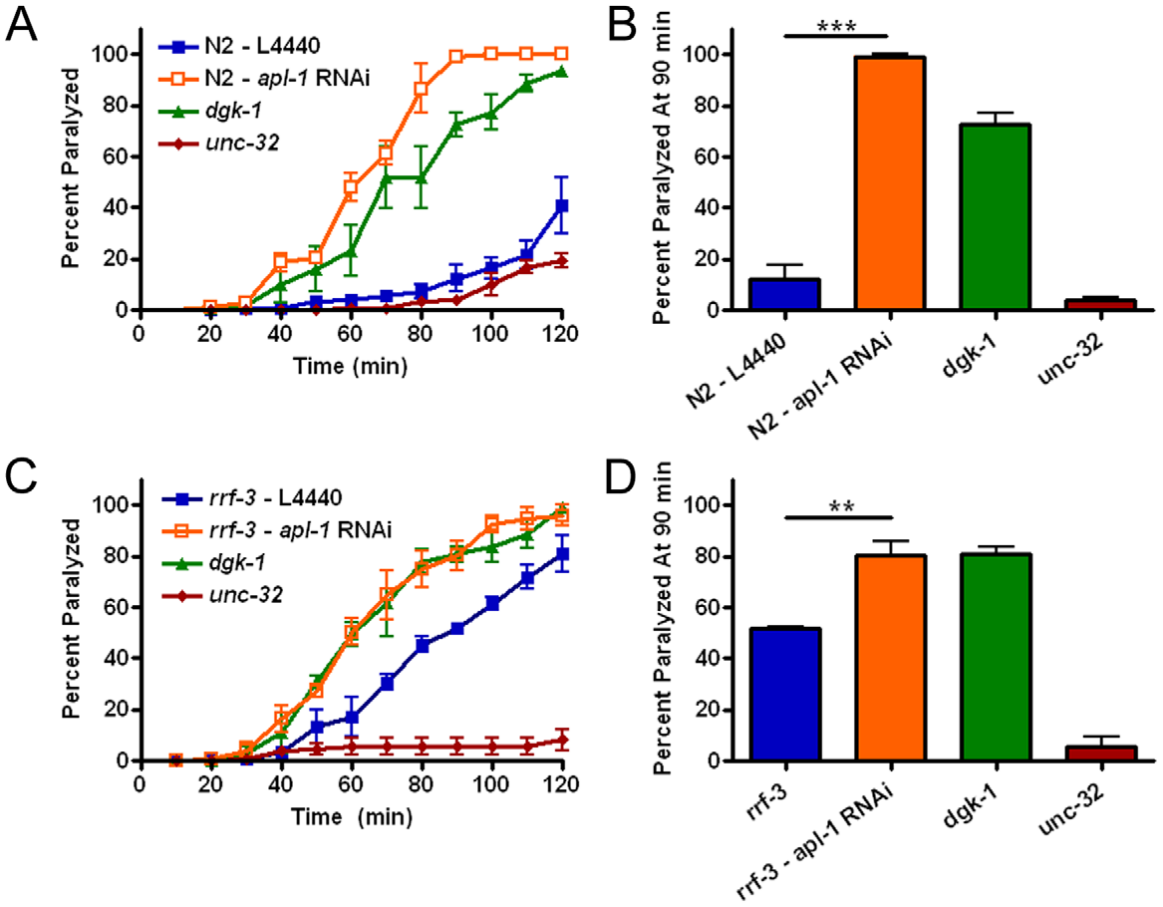
Aldicarb hypersensitivity from loss of *apl-1*. (A) Aldicarb sensitivity over time from worms exposed to *apl-1* RNAi throughout development. *apl-1* knock-down leads to aldicarb hypersensitivity. Controls: *dgk-1* - diacylglycerol kinase mutant, aldicarb hypersensitive; *unc-32* - mutant for a vacuolar H^+^-ATPase, aldicarb resistant. (B) Quantification at the 90 minute time point. (C) To avoid the molting defect, adult worms were placed on *apl-1* RNAi, which also led to the same aldicarb hypersensitivity. (D) Quantification of the adult RNAi aldicarb experiment at 90 minutes. Each experiment was performed at least three times (n = 50 per strain). (**, P<0.01; ***, P<0.001) All error bars represent the s.e.m.

Mammalian studies have revealed that mice lacking both APP and APLP2 display impaired synaptic structure and function at the peripheral cholinergic neuromuscular junction [24]. To examine whether *apl-1* knock-down has a similar effect on neurotransmission, worms were tested for their response to the acetylcholinesterase inhibitor aldicarb, which blocks the breakdown of acetylcholine in the synaptic cleft, leading to constant stimulation of postsynaptic receptors and paralysis over time [13], [25]. Worm mutants with excess or depleted acetylcholine become hypersensitive or resistant to aldicarb respectively. We found that *apl-1* RNAi treated worms exhibited hypersensitivity to aldicarb (Figure 2A, B). In order to address the question of whether this phenotype may be a secondary effect due to the molting defect also seen in these worms, we bypassed the molting stages by placing RNAi sensitive young adult worms that have completed the molt cycle on *apl-1* RNAi and subjected them to aldicarb testing. These worms were also hypersensitive to aldicarb (Figure 2C, D), indicating a direct effect of APL-1 on neurotransmission, independent of molting.

**Figure 3:**
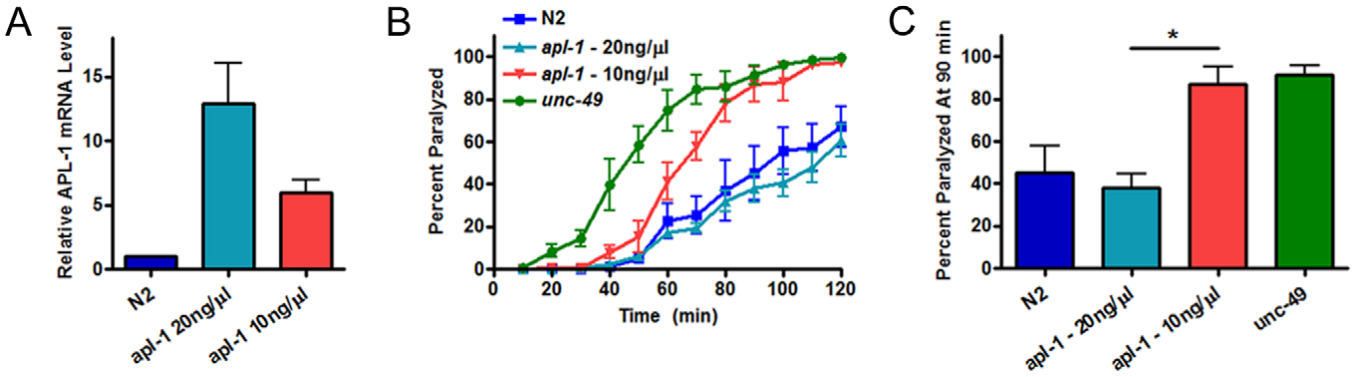
Rescue of aldicarb hypersensitivity by *apl-1* is dose dependent. (A) qRT-PCR showing representative rescue strain expression of *apl-1* at different injection concentrations. Error bars represent the STD. (B) Aldicarb hypersensitivity is not rescued by *apl-1* full length at a lower expression level. Strains expressing half the level of the original full length strain can no longer rescue the aldicarb hypersensitivity, but rather show a dosage dependent effect. Two rescue strains per genotype were used to rule out variability from over-expression of the rescue array (representative strains shown). Control: *unc-49* - GABA receptor subunit, aldicarb hypersensitive. (C) Quantification of the rescue strain aldicarb experiment at 90 minutes. (*, P<0.05) Error bars represent the s.e.m. unless otherwise noted.

Next, we examined whether the transgenically expressing full length APL-1 rescue strains were able to rescue this neurotransmission defect. We tested full length strains made by injecting different concentrations of DNA (20 ng/µl and 10 ng/µl), and therefore having different levels of transgenic protein expression confirmed by both qRT-PCR and Western blot (Figure 3A, S4A). Interestingly, only the strain that expressed the higher concentration of APL-1 was able to rescue the aldicarb hypersensitive phenotype while the lower expressing strain did not (Figure 3B, C). These results show a dosage dependent effect of APL-1. This effect was not due to the presence of the GFP tag, as rescue strains with a similar level of expression without the tag retained the aldicarb hypersensitivity (Figure S3). Since the rescue of aldicarb hypersensitivity, but not molting or development, by full length APL-1 is dose dependent, this result further strengthens the notion that APL-1 directly mediates synaptic transmission independent of molting.

To test whether the C-terminus of *apl-1* is required for the regulation of neurotransmission, we tested the aldicarb sensitivity of the ΔIC and the ΔYENPTY rescue strains and found that the C-terminal mutants exhibited similar aldicarb hypersensitivity as compared to full length APL-1 expressed at comparable levels (Figure S4). These data provide indirect support against a potent role of the highly conserved C-terminal domain in APL-1 mediated synaptic transmission.

### UNC-104/KIF1A, UNC-116/Kinesin 1 and RAB-5 Positively Regulate APL-1 Expression

**Figure 4:**
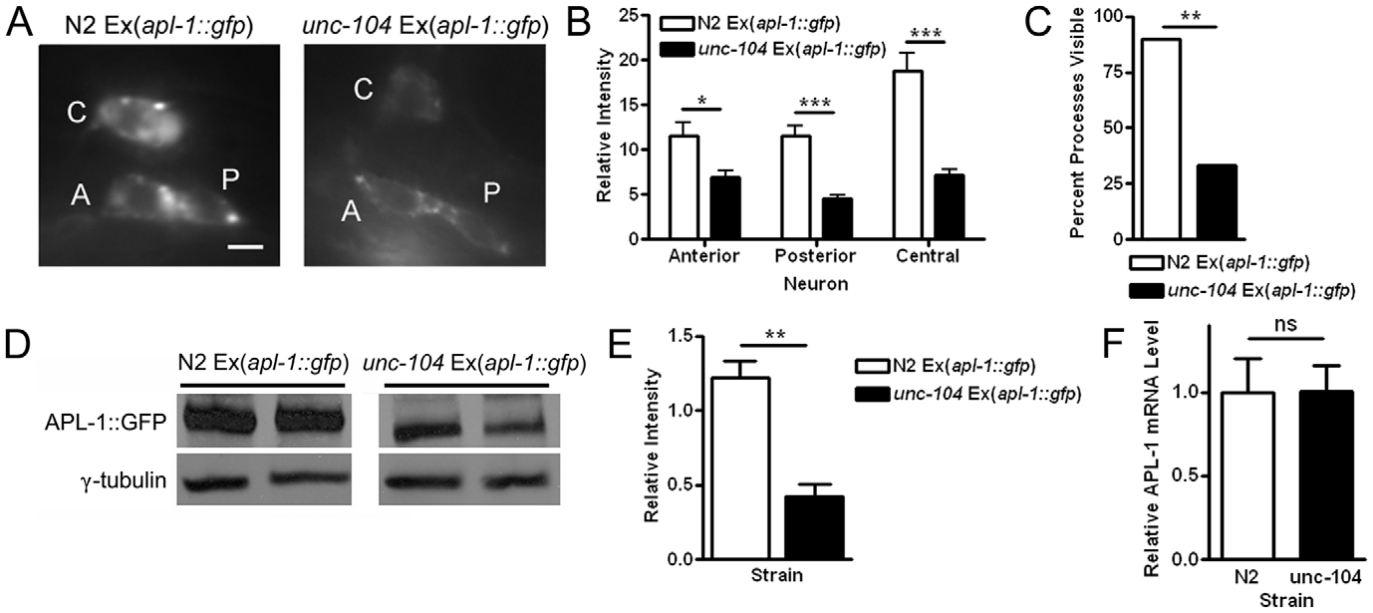
Loss of the neuronal kinesin *unc-104* leads to a decrease of APL-1 expression. (A) Representative pictures of head interneurons expressing *apl-1::gfp* on N2 and *unc-104* backgrounds. Neurons are arbitrarily labeled A, P and C for Anterior, Posterior and Central respectively. (Scale bar, 2.5 µm) (B) Quantification of APL-1::GFP fluorescence in the cell bodies of the corresponding head neurons (n = 20 per strain). (C) Percent of dorsal cord processes derived from a single head neuron visible in the N2 and *unc-104* backgrounds (N2 = 10, *unc-104* = 15). (D) Western blot showing diminished APL-1 protein levels in the kinesin mutant. Two lines per genotype were used for analysis. (E) Quantification of Western blot. (F) qRT-PCR data showing unchanged transcription levels of *apl-1* between the N2 and *unc-104* mutant. Error bars represent the STD. (*, P<0.05; **, P<0.01; ***, P<0.001) Error bars represent the s.e.m. unless otherwise noted.

While the trafficking of APP has been extensively studied in neurons, the movement of APL-1 through the cell is still unknown. APP is normally trafficked in a kinesin-dependent anterograde fashion from the cell body to the nerve terminal [26]. Due to APL-1's strong expression in neurons, we decided to test whether APL-1 can be transported by two of the major kinesins involved in anterograde transport of synaptic proteins. The first kinesin we investigated is the worm homolog of KIF1A, UNC-104. UNC-104/KIF1A is responsible for transporting synaptic vesicles and dense core vesicles to sites of synaptic transmission [27], [28], [29]. In order to test if APL-1 transport depends on this neuronal kinesin, we crossed the APL-1::GFP transgenic rescue strain to the hypomorphic mutant *unc-104(e1265)*. Interestingly, rather than observing an accumulation of the GFP fluorescence in the cell body, which traditionally results from the reduction of UNC-104 mediated vesicle transport [27], we found a dramatic decrease in the fluorescence from *apl-1::gfp* on the *unc-104(e1265)* background, as measured by fluorescence intensity in a set of three head inter-neurons that consistently expressed *apl-1* (Figure 4A, B) Additionally, APL-1 fluorescence was absent from the processes of the neurons, quantified from a specific dorsal process that is consistently visible on the N2 background (Figure 4C). Western blotting from L4 worms also detects a drop in APL-1 protein expression on the *unc-104(e1265)* background (Figure 4D, E). qRT-PCR comparing *apl-1* expression between N2 and *unc-104(e1265)* backgrounds showed no differences, suggesting that the loss of APL-1::GFP is occurring at the protein level (Figure 4F).

Using a similar approach, we tested the ability of UNC-116/kinesin-1 to transport APL-1 by crossing the APL-1::GFP transgenic strain to the hypomorphic *unc-116(e2310)* background. Kinesin-1 has been found to play a prominent role in the transport of APP along the axon [26]. Again we saw a reduction in the amount of APL-1::GFP expression on the *unc-116* mutant background, although to a lesser extent as that on the *unc-104* background (Figure S5). However, there was a complete loss of APL-1 expression along the dorsal axon in every nematode analyzed on the *unc-116* background. These results suggest that APL-1 localization is dependent on both kinesins.

**Figure 5:**
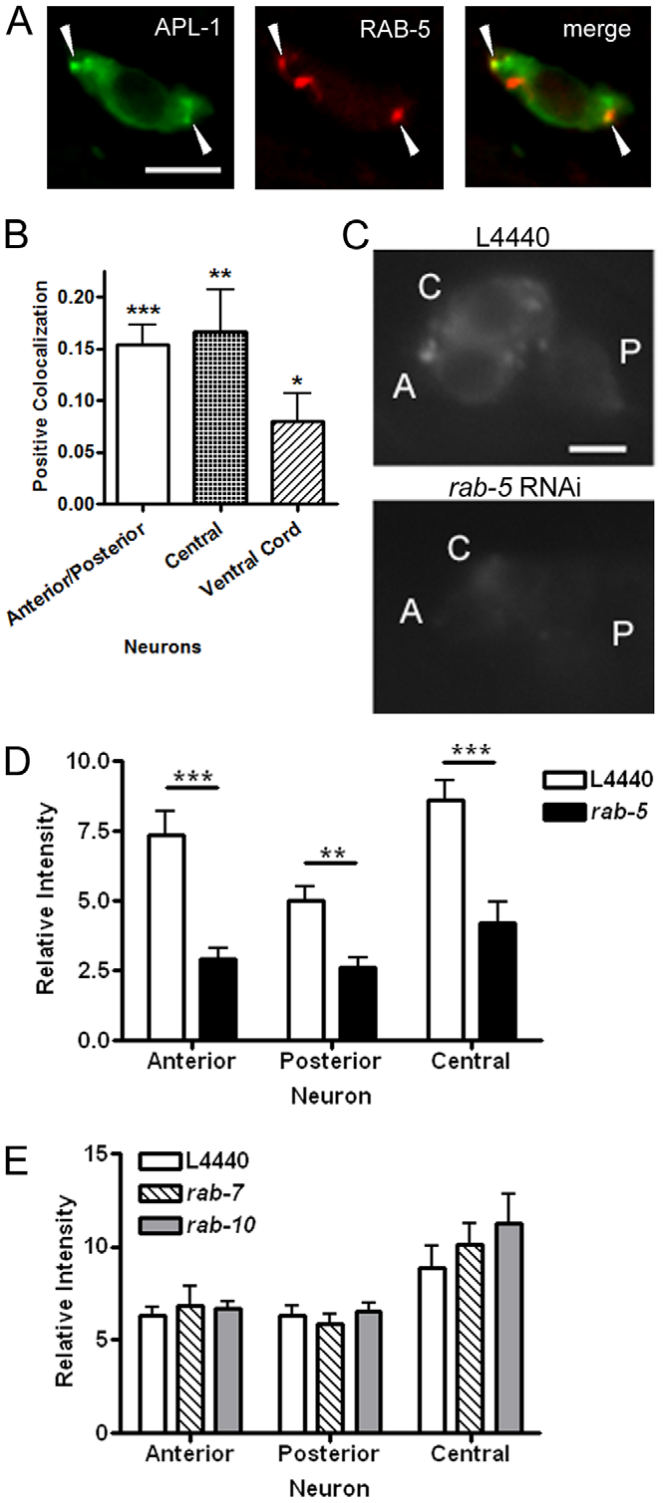
Knock-down of the early endosomal small GTPase *rab-5* leads to a loss of APL-1::GFP. (A) Colocalization of APL-1::GFP and mCherry::RAB-5. Arrowheads highlight regions of most intense overlap. (Scale bar, 2.5 µm) (B) Colocalization analysis showing positive colocalization between APL-1 and UNC-108 in all neuronal populations examined. Values depicted are an index of the association of staining intensities of the two fluorophores known as the intensity correlation quotient. Positive values indicate colocalization while a value of zero indicates random staining. Values were compared statistically to zero (random staining). (C) Representative pictures depicting the cell bodies of the head neurons of *apl-1::gfp* expressing worms either on control bacteria (L4440) or *rab-5* RNAi. (Scale bar, 2.5 µm) (D) Quantification of APL-1::GFP fluorescence in the neuronal cell bodies of worms on L4440 or *rab-5* RNAi (n = 10). (E) Quantification of APL-1::GFP fluorescence on control, *rab-7* and *rab-10* RNAi (n = 10). No difference in fluorescence intensity was seen during *rab-7* or *rab-10* knock-down. (*, P<0.05; **, P<0.01; ***, P<0.001) Error bars represent the s.e.m.

In addition to being transported by kinesins, APP has previously been shown to localize to the early endosome by its strong colocalization with Rab5 positive compartments in preparations of nerve terminals from rat forebrain and PC12 cells [30], [31]. As a small GTPase, Rab5 regulates endosomal trafficking of vesicles from the plasma membrane to the early endosome [32]. To determine whether APL-1 was also present in RAB-5 compartments in the worm we generated strains co-expressing APL-1::GFP and mCherry::RAB-5. We found that these proteins colocalized within a subset of puncta (Figure 5A). This was reconfirmed by observing consistently positive intensity correlation quotient (ICQ) values in the different neurons analyzed (Figure 5B).

To determine whether loss of *rab-5* had an effect on the localization of APL-1, we used RNAi to knock-down *rab-5* expression in an integrated APL-1::GFP expression strain on the RNAi sensitive *rrf-3(pk1426)* background. Loss of *rab-5* led to a dramatic decrease in the amount of APL-1::GFP in neurons as well as a complete loss of APL-1 in the dorsal process (Figure 5C, D). By contrast, knock-down of two other small GTPases, *rab-7* or *rab-10*, did not affect APL-1 expression, suggesting that RAB-5 compartments specifically are important for the localization of APL-1 (Figure 5E).

### *unc-108* Mutations Lead to Altered Intracellular Localization of APL-1

UNC-108 is a small GTPase expressed in neurons and engulfing cells that localizes to the Golgi and early endosome [33], [34]. UNC-108 has been found to be involved in the maturation of dense core vesicles (DCVs), a distinct vesicular population containing peptide hormones and neuropeptides [34], [35], [36]. APL-1 likely undergoes fast axonal transport in a vesicular population, therefore we wanted to investigate if UNC-108 is required for the packaging of APL-1 into vesicles destined for anterograde transport.

**Figure 6:**
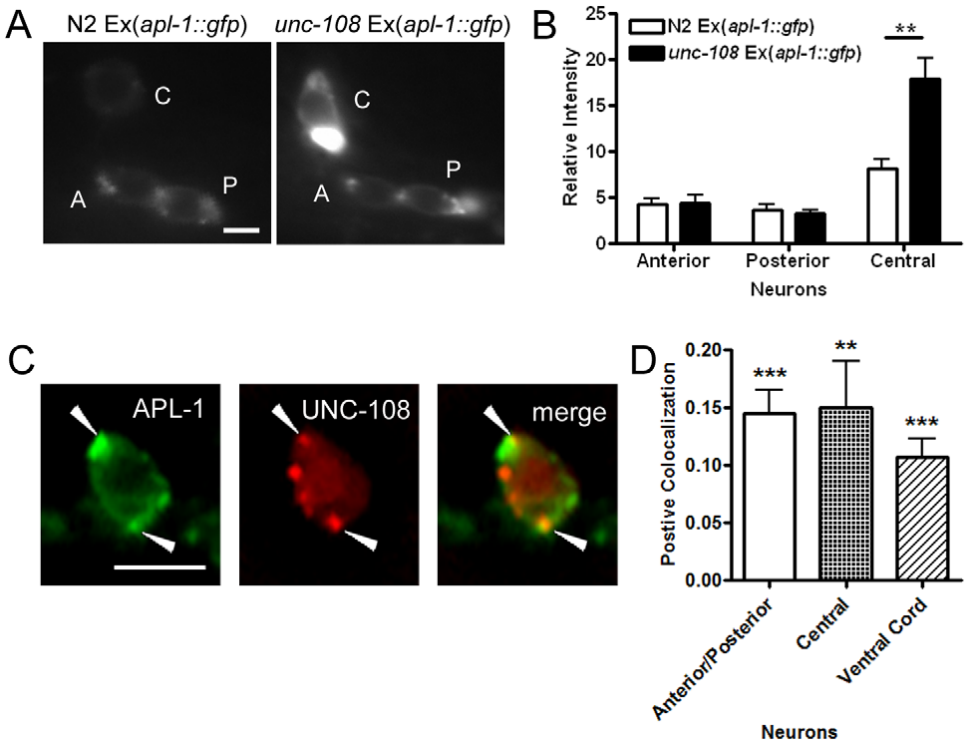
UNC-108 regulates APL-1 localization. (A) Representative pictures of the back-up of APL-1::GFP in the cell body of the central interneuron in the hypomorphic *unc-108(n3263)* mutant. (Scale bar, 2.5 µm) (B) Quantification of APL-1::GFP fluorescence in N2 and *unc-108(n3263)* neuronal cell bodies (n = 10). (C) Colocalization of APL-1::GFP with mCherry::UNC-108. Arrowheads point to overlapping puncta. (Scale bar, 2.5 µm) (D) Colocalization analysis showing a positive colocalization intensity correlation quotient under every neuronal population quantified. (**, P<0.01; ***, P<0.001) Error bars represent the s.e.m.

To study this possibility we crossed the hypomorphic mutant *unc-108(n3263)* to the APL-1::GFP expressing strain. We found that one of the head inter-neurons appeared to have a back-up of protein in the cell body (Figure 6A, B). This aggregation of protein in a distinct compartment was also seen in ventral cord neurons. We then performed colocalization experiments by generating strains expressing APL-1::GFP and mCherry::UNC-108. Similar to the colocalization with RAB-5, APL-1 is found in overlapping puncta with UNC-108 in neurons, demonstrating APL-1 localization to the same compartment (Figure 6C). The ICQ values of the different neuronal populations were consistently positive, showing that the proteins colocalize together at a similar frequency as with RAB-5 (Figure 6D). These data suggest that UNC-108 is required for the localization of APL-1 and ultimately its transport.

## Discussion

### N-terminus of APL-1 Is Required for Worm Survival and Molting

We have shown that loss of *apl-1* contributes to defects in at least two systems, one of which is molting. The L1 lethality seen in the *apl-1*(*tm385*) strain is likely due, at least in part, to the molting defect, which is recapitulated by *apl-1* RNAi. Worms with *apl-1* knocked-down share the loose cuticle and internal vacuolization phenotypes of *apl-1(tm385)* L1s but exhibit these phenotypes later in development. The clear appearance of these worms may be due to inappropriate release of proteases involved in the molting process, loss of proper adhesion between tissues, and/or loss of fat stores due to starvation from the loose cuticle blocking food intake. Whether the sluggish movement defect seen in adult *apl-1* knock-down worms is due to the molting defect, starvation or a neurotransmission defect remains to be seen. The phenotypes shown in this RNAi study are more severe than those described previously [37], possibly due to our use of an RNAi sensitive strain to ensure neuronal knock-down of *apl-1*.

The particular molting defect seen with *apl-1* RNAi is indicative of a failure to undergo ecdysis, or shedding of the old exoskeleton [38]. Another single-pass trans-membrane protein possessing a similar loose cuticle molting defect is the LDL receptor-related protein (LRP-1), which is the *C. elegans* ortholog of LRP-2/megalin and likely functions in cholesterol uptake and homeostasis [38], [39], [40]. A null mutation in LRP-1, like APL-1, leads to arrest and lethality although at later larval stages [40]. These similarities suggest that *lrp-1* and *apl-1* may operate in the same or similar pathways to control the molting process. Since we found that APL-1 does not require its C-terminal domain for rescue of the molting defect and soluble, secreted APP has been shown to bind to an LRP homolog [41], an attractive hypothesis would be that the N-terminal domain of APL-1 is shed and released at regulated periods followed by binding to LRP-1 to mediate proper ecdysis at each of the four molts.

Our rescue results and previous studies support the notion that only the N-terminus of APL-1 is required to rescue the lethality seen in the *apl-1* null strain [8]. In mice, expression of APP that has been truncated either at the α-cleavage site or had the last 15 amino acids removed could ameliorate APP knock-out phenotypes such as reduced body and brain weight, defective LTP and spatial learning, and loss of grip strength [42]. These findings combined support an important function of the N-terminus of APP in the mammalian system as well as in *C. elegans*.

While the behavior defects in the *Drosophila* APPL null can be rescued by expression of human APP [43], we were unable to rescue the *apl-1* null lethality in *C. elegans* by expressing any of the human homologs of APP. Several notable differences between the fly and the worm homolog of APP could account for the differences in cross species rescue. Unlike APL-1, expression of the fly homolog is confined to neurons and loss of APPL does not affect Drosophila viability or fertility [43], [44]. Furthermore, different domains in APL-1 and APPL are required for their respective functions. The entire APPL protein is required for its proper function in the fly, whereas in *C. elegans* the APL-1 N-terminus is the critical domain needed to rescue the lethal *apl-1* null mutant. APPL over-expression induced synaptic bouton formation could be prevented by deletion of the C-terminal domain or the N-terminal E1 and E2 domains, showing that the holoprotein is needed to mediate this function [45]. That APP with a C-terminal truncation expressed in APPL deficient fly lines could no longer induce axonal arborization seen when expressing full length APP further highlights the importance of the C-terminus for proper function of the protein in flies [46]. Since APL-1 function requires the N-terminus, it is not surprising that the *C. elegans* system cannot use APP with its minimally conserved N-terminal domains to rescue the severe *apl-1* loss-of-function phenotypes.

### Loss of *apl-1* Leads to Neurotransmission Defects

*C. elegans* and mice share many homologs that are involved in synaptic structure and function. Therefore we predicted that *C. elegans* would be an excellent model to study the importance of APL-1 in neurotransmission. In mice, *APP/APLP2* null animals have enhanced nerve sprouting, reduced numbers of synaptic vesicles, defects in neurotransmitter release as well as a large number of defective synapses [24]. Similar to the mammalian system, we reveal here that loss of *apl-1* expression leads to defective neurotransmission. We did not observe any overt defect in general neuronal structure in *apl-1(tm385)* lethal L1s, therefore defects in the development of the neuronal network are not likely to contribute to the phenotype. Interestingly, we would predict that the defect on aldicarb would be resistance rather than hypersensitivity if the worms lacking APL-1 also have a reduction in synaptic vesicle number and decreased number of functional synapses. These differences may be due to the fact that the mammalian system uses purely cholinergic connections at the neuromuscular junction while worm movement is modulated by both GABAergic and cholinergic synapses. Aldicarb cannot distinguish between cholinergic or GABAergic defects, nor can we rule out contributions from dense core vesicles, which also modulate neurotransmission [47], [48], [49]. The hypersensitivity we see during *apl-1* knock-down may be due to defects in some or all of these systems. Future studies will address whether the number and/or internal structure of the synapses in each of these systems are affected by loss of *apl-1*. However, we predict that defects found in synaptic number or structure, if any, will be subtle due to the lack of profound locomotor defects during *apl-1* knock-down or in the *apl-1* loss-of-function mutant.

Another prospective pathway APL-1 may use to mediate synaptic transmission is the well studied EGL-30 G-protein coupled receptor pathway, which can modulate cholinergic signaling. Loss-of-function mutations in negative regulators of this G_q_α pathway including *goa-1*/G_o_α, *eat-16*/RGS7 and *dgk-1*, all lead to a hypersensitive phenotype on aldicarb [50]. In addition, APL-1 and APP share a conserved G_o_ protein binding domain on its C-terminus [8], [51]. While the C-terminus of APL-1 may not be required for performing proper molting, we cannot rule out an important role for this domain in neurotransmission since we could not create a strain that rescues the aldicarb hypersensitivity with the ΔIC construct. Also, the dosage dependent effect we see with full length APL-1 may be due to improper regulation of this pathway due to varying levels of interaction with G_o_. The fact that loss of *apl-1* also leads to enhanced pharyngeal pumping [52] supports a regulatory role through the EGL-30 pathway as pharyngeal pumping is one of the many functions modulated by this G-protein [53].

We have found that the regulation of neurotransmission by APL-1 does not appear to be related to its regulation of molting. A molting defect was not seen in any of the rescue strains, or purposely avoided by performing *apl-1* RNAi knock-down in adults, whereas the aldicarb hypersensitivity was present in these worms. This dual regulation could not be dissected by removing the C-terminal domain or YENPTY motif, since the full length APL-1 rescue of lethality at a lower expression level still could not rescue the aldicarb hypersensitivity. Together, these data support a model in which the function of APL-1 in molting is independent of its function in neurotransmission.

### Regulation of APL-1 Localization and Transport

**Figure 7:**
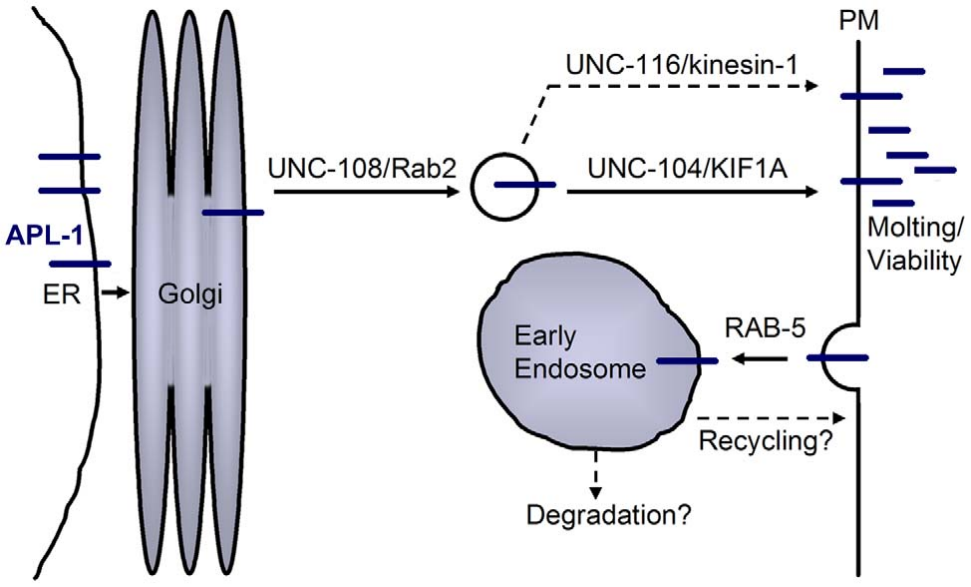
Model for APL-1 transport and function. As a transmembrane protein, APL-1 moves through the endoplasmic reticulum (ER) and Golgi followed by sorting into its appropriate vesicular population through the action of UNC-108. From there, proper APL-1 transport through the neuron depends on the motor proteins UNC-104 or UNC-116. Once APL-1 reaches the plasma membrane (PM) it can be endocytosed and transported to the early endosome through the action of RAB-5. APL-1 is then likely sent for degradation or recycled back to the plasma membrane. While APL-1 processing is unclear, release of the extracellular domain is necessary for survival and molting. Whether APL-1 is able to regulate neurotransmission at the plasma membrane or at some point upstream has yet to be determined.

We have found that localization of APL-1 in neurons is regulated through the action of the kinesins UNC-104/KIF1A and UNC-116/kinesin-1 as well as the small GTPases RAB-5 and UNC-108/Rab2 (Figure 7). In mice, APP undergoes fast axonal transport to the nerve terminal through the action of the kinesin-1 transporter [26]. However, in worms, APL-1 localization is dependent on both UNC-104/KIF1A and UNC-116/Kinesin-1. Rather than causing a back-up of protein, loss of either of these kinesins led to a general loss of APL-1::GFP. This indicates that the protein is being broken down rather than being allowed to accumulate in the cell body. Neither of these hypomorphic mutants have a molting defect, possibly through compensation of APL-1 trafficking by the other transporter, or the decrease in function of the kinesin is not severe enough to prevent APL-1 from operating in the molting pathway.

After being exposed to the cell surface, APP is rapidly internalized and sorted to the early endosome by the action of the small GTPase Rab5 [31]. Like APP, we have found that APL-1 movement through the endosomal pathway utilizes RAB-5 as loss of RAB-5 reduces the level of APL-1 within the neuron. With the loss of RAB-5 through RNAi, we speculate that APL-1 becomes trapped on the cell surface where it is subject to increased exposure to proteases on the plasma membrane, which may account for the diffusion of GFP signal.

UNC-108/Rab2 is known for its role in COPI-mediated retrograde transport between the Golgi and ER [54]. However, in *C. elegans*, loss of *unc-108* does not affect COPI transport, but rather leads to an accumulation of early endosomal compartments [55]. UNC-108 is also involved in the maturation of dense core vesicles by preventing loss of cargo to specific endosomal compartments [34], [36]. We suspect that APL-1 accumulation in *unc-108(n3263)* mutants may be due to incorrect sorting of APL-1 into the proper vesicular population upstream of anterograde transport. It is still unknown whether APL-1 is present in dense core vesicles, although this subset of vesicles is primarily transported by UNC-104 [56], which also appears to be the transporter most involved in proper localization and expression levels of APL-1 (Figure 4). Since the localization of APL-1 is dependent upon the presence of functional UNC-108, and the two proteins colocalize, it is possible that APL-1 may play a role either within the DCVs, or actively operating with UNC-108 in the maturation of DCVs. This may be another plausible explanation for the ability of APL-1 to regulate synaptic transmission as dense core vesicle cargos have been found capable of modulating cholinergic signaling [49].

In summary, our results show that APL-1 regulates neurotransmission independently of its function in the molting process. APL-1 moves through the neuron in a similar fashion to APP, with the distinction that two kinesins are needed for anterograde transport and to maintain proper expression levels of APL-1. Like APP, this transport is followed by endocytosis through the action of RAB-5. The ability of UNC-108 to alter the localization of APL-1 points to a novel process by which APL-1 is regulated in the cell. Overall, we predict that transport of APL-1 within the neuron enables APL-1 to properly perform its multiple functions by introducing the protein to molecules that can cleave and regulate release of the critically important N-terminal portion of the protein. This has implications for the biology of APP and its homologs where the N-termini of these proteins may also act as ligands to stimulate downstream pathways that modulate neurotransmission.

## Supporting Information

File S1 Supplementary tables.
(0.07 MB DOC)

Figure S1 Molting defect seen during *apl-1* RNAi. (A) Representative pictures of the various molting defects seen in RNAi sensitive strain *rrf-3(pk1426)* L4s during *apl-1* knock-down. Arrowheads point to regions described by the inlaid text. (Scale bar, 20 µm.) (B) Percentages of the different molting defects seen in worms on *apl-1* RNAi after 48 hours from egg stage (n = 173). (C) Percentage of different progeny stages 48 hours from egg stage on both control (L4440) and *apl-1* RNAi.
(1.06 MB TIF)

Figure S2 Rescue strains of *apl-1 (tm385)* have slower development. (A) Map of *apl-1(tm385)* deletion and premature stop codon. (B) Percentage of different larval stages after 48 hours of growth. The N2 strain and heterozygous *apl-1(tm385)/lon-2(e678)* are shown as negative controls. (N2, n = 697; *apl-1/lon-2*, n = 186; N2 Ex(*apl-1::gfp*)- 20 ng/µl, n = 528). Missing worms were those unaccounted for after 48 hours from the original total of eggs placed on the plate. These are likely in large proportion part of the L1 lethal population that are no longer visible. (C) No movement defects were detected in the transgenic *APL-1::GFP* strain. (N2, n = 14; *apl-1/lon-2*, n = 10; N2 Ex(*apl-1::gfp*)- 20 ng/µl, n = 14). (D) Brood sizes from the transgenic *APL-1::GFP* strain were normal when compared to the N2 and *apl-1/lon-2* strains. (N2, n = 14; *apl-1/lon-2*, n = 11; N2 Ex(*apl-1::gfp*) - 20 ng/µl, n = 14). (*, P<0.05; **, P<0.01; ***, P<0.001.) Error bars represent the s.e.m.
(0.64 MB TIF)

Figure S3 C-terminal GFP is not detrimental to the full length APL-1 rescue. (A) qRT-PCR of rescue strains showing comparative APL-1 expression with or without GFP. Error represents the STD. (B) Aldicarb experiment showing the retained hypersensitivity of full length APL-1 without the GFP tag at 10 ng/µl and 5 ng/µl. Error is the s.e.m.
(0.28 MB TIF)

Figure S4 C-terminal mutation rescue strains cannot rescue the aldicarb hypersensitivity. (A) Western blot showing protein expression levels from the different rescue strains. (B) Rescue strains with either a C-terminal truncation or deletion of the YENPTY domain could not rescue the aldicarb hypersensitivity. (C) Quantification of the aldicarb experiment at the 90 min time-point. (*, P<0.05; **, P<0.01.) Error bars represent the s.e.m.
(0.54 MB TIF)

Figure S5 Loss of kinesin-1 function also leads to a reduction in APL-1::GFP fluorescence. (A) Representative pictures of head neurons with *apl-1::gfp* expression on N2 and *unc-116* backgrounds. (Scale bar, 2.5 µm.) (B) Quantification of APL-1::GFP fluorescence (n = 10 per strain). (*, P<0.05.) Error bars represent the s.e.m.
(0.41 MB TIF)
