# Supplementary material for: Intracellular Trafficking and Synaptic Function of APL-1 in Caenorhabditis elegans
Source: PLoS One. 2010 Sep 20;5(9):e12790. doi: 10.1371/journal.pone.0012790 (PMC2942829; doi:10.1371/journal.pone.0012790)
Supplement: File S1 — Supplementary tables. (0.07 MB DOC) [file pone.0012790.s001.doc]

**Supplementary Tables**

**Supplementary Table 1. Cloning primers used for construct generation in this study.**

| **Construct Name** | **Primer Name** | **Primer Sequence (5’ to 3’)** |
| --- | --- | --- |
| *papl-1::apl-1::gfp* | papl-1 Fwd XbaI | CGTA TCTAGA CAACGTTCTCATCTTTCAATTCCAG |
|  | apl-1 Rev NotI | GTAC GCGGCCGC GGCCTTCGAGTCGAAGAATGAGTAC |
| *papl-1::apl-1* | apl-1 Rev NotI | GTAC GCGGCCGC GGCCTTCGAGTCGAAGAATGAGTAC |
|  | papl-1 Fwd XbaI | CGTA TCTAGA CAACGTTCTCATCTTTCAATTCCAG |
|  | L3781 MCS XbaI | CGTA TCTAGA GTCGACCTGCAGGCATG |
|  | L3781 End NotI | CGTA GCGGCCGC TGGAATTCGCTAGCCGGCC |
| *papl-1::apl-1ΔYENPTY::gfp* | YENPTY Fwd | GCTGGAATGCAAGTCAATGGCTCATTCTTCGACTCGAAGGCC |
|  | YENPTY Rev | GGCCTTCGAGTCGAAGAATGAGCCATTGACTTGCATTCCAGC |
| *papl-1::apl-1T658A::gfp* | T658A Fwd | CATCGAGGTAGACGTCTACGCACCAGAGGAGCGTCATGTCG |
|  | T658A Rev | CGACATGACGCTCCTCTGGTGCGTAGACGTCTACCTCGATG |
| *papl-1::apl-1T658E::gfp* | T658E Fwd | CATCGAGGTAGACGTCTACGAACCAGAGGAGCGTCATGTCG |
|  | T658E Rev | CGACATGACGCTCCTCTGGTTCGTAGACGTCTACCTCGATG |
| *papl-1::apl-1ΔIC::gfp* | apl-1 EX Rev KpnI | CGTA GGTACC ATCATGGCACGGCGACGACGAGC |
|  | apl-1 Fwd NotI | CGTA GCGGCCGC ATGACGGTGGGTAAACTAATGATTGGC |
|  | papl-1 Fwd XbaI | CGTA TCTAGA CAACGTTCTCATCTTTCAATTCCAG |
|  | papl-1 Rev NotI | CGTA GCGGCCGC GGCTGAAAAAATGTCTGAATTAG |
| *papl-1*::APP::RFP | APP Fwd XmaI | CGTA CCCGGG ATGCTGCCCGGTTTGGCACTGC |
|  | RFP Rev (NheI) | GTAC GCTAGC TTAGGCGCCGGTGGAGTGGCG |
|  | papl-1 Fwd SacII | GTAC CCGCGG GAACGCCCTCCAACGTTCTCATC |
|  | papl-1 Rev XmaI | CGTA CCCGGG GGCTGAAAAAATGTCTGAATTAG |
| *papl-1*::APLP1::GFP | APLP1 Fwd SacII | GTAC CCGCGG ATGGGGCCCGCCAGCCCCGCTG |
|  | APLP1 Rev KpnI | GTAC GGTACC CAGGGTCGTTCCTCCAGGAAGCGG |
|  | papl-1 Fwd XbaI | CGTA TCTAGA CAACGTTCTCATCTTTCAATTCCAG |
|  | papl-1 Rev NotI | CGTA GCGGCCGC GGCTGAAAAAATGTCTGAATTAG |
| *papl-1*::APLP2::GFP | APLP2 Fwd XmaI | GTAC CCCGGG ATGGCGGCCACCGGGACCGCGG |
|  | APLP2 Rev KpnI | GTAC GGTACC CTAATCTGCATCTGCTCCAGGTATTTGTAG |
|  | papl-1 Fwd XbaI | CGTA TCTAGA CAACGTTCTCATCTTTCAATTCCAG |
|  | papl-1 Rev XmaI | CGTA CCCGGG GGCTGAAAAAATGTCTGAATTAG |
| *papl-1::*mCherry::*rab-5* | papl-1 Fwd XbaI | CGTA TCTAGA CAACGTTCTCATCTTTCAATTCCAG |
|  | papl-1 Rev XmaI b | CGTA CCCGGG TCATTAGTTTACCCACCGTCATGGC |
|  | mCherry XmaI Fwd | CGTA CCCGGG ATGGTCTCAAAGGGTGAAGAAG |
|  | mCherry NcoI Rev | CGTA CCATGG CAGC TACAATTCCTCCATGCCAC |
| *punc-108::*mCherry*::unc-108* | mCherry PstI Fwd | CGTA CTGCAG ATGGTCTCAAAGGGTGAAGAAG |
|  | mCherry XmaI Rev | CGTA CCCGGG CTAATACAATTCCTCCATGCCACC |
|  | unc-108 L65Q Fwd | GGGACACAGCCGGACAAGAATCATTCCGCTCC |
|  | unc-108 L65Q Rev | GGAGCGGAATGATTCTTGTCCGGCTGTGTCCC |

**Supplementary Table 2. Transgenic worm strains generated in this study.**

| **Strain Name** | **Genotype** | **DNA Concentration** |
| --- | --- | --- |
| AA1435 | N2 dhEx535[*papl-1::apl-1::gfp*] | 40ng/μl |
| AA1445 | *apl-1(tm385)* dhEx544[*papl-1::apl-1::gfp*] | 20ng/μl |
| AA1493 | *apl-1(tm385)* *dhEx598*[*papl-1::apl-1::gfp*] | 10ng/ul |
| AA1538 | *apl-1(tm385)* *dhEx607*[*papl-1::apl-1*] 20ng/ul | 20ng/μl |
| AA1537 | *apl-1(tm385)* *dhEx606*[*papl-1::apl-1*] 10ng/ul | 10ng/ul |
| AA1539 | *apl-1(tm385)* *dhEx608*[*papl-1::apl-1*] 5ng/ul | 5ng/μl |
| AA1504 | N2 *dhEx544*[*papl-1::apl-1::gfp*] | 20ng/μl |
| AA1486 | *apl-1(tm385)* *dhEx594*[*papl-1::apl-1ΔIC::gfp*] | 10ng/ul |
| AA1443 | *apl-1(tm385*) *dhEx542*[*papl-1::apl-1ΔYENPTY::gfp*] | 20ng/μl |
| AA1516 | *apl-1(tm385)* *dhEx605*[*papl-1::apl-1 T658E::gfp*] | 20ng/μl |
| AA1510 | *apl-1(tm385)* *dhEx603*[*papl-1::apl-1 T658A ::gfp*] | 20ng/μl |
| AA1440 | *apl-1(tm385)/lon-2(e678)* *dhEx539*[*papl-1::hAPP::rfp*] | 20ng/μl |
| AA1447 | *apl-1(tm385)/lon-2(e678)* *dhEx546*[*papl-1::APLP1::gfp*] | 20ng/μl |
| AA1449 | *apl-1(tm385)/lon-2(e678)* *dhEx548*[*papl-1::APLP1::gfp*] | 10ng/ul |
| AA1451 | *apl-1(tm385)/lon-2(e678)* *dhEx563*[*papl-1::APLP2::gfp*] | 20ng/μl |
| AA1482 | *apl-1(tm385)/lon-2(e678)* *dhEx591*[*papl-1::hAPP::rfp; papl-1::hAPLP1::gfp; papl-1::hAPLP2::gfp*] | 20ng/μl each |
| AA1499 | *unc-104(e1265)* *dhEx544*[*papl-1::apl-1::gfp*] | 20ng/μl |
| AA1527 | *unc-116(e2310)* *dhEx544*[*papl-1::apl-1::gfp; pmyo-3::cfp*] | 20ng/μl |
| AA1503 | N2 *dhEx601*[*papl-1::apl-1::gfp; papl-1::mCherry::rab-5*] | 20ng/μl each |
| AA1513 | *apl-1(tm385)*;*rrf-3(pk1426)* *dhIs504*[*papl-1::apl-1::gfp*] | 10ng/ul |
| AA1540 | *unc-108(n3263)* *dhEx544*[*papl-1::apl-1::gfp*] | 20ng/μl |
| AA1550 | N2 *dhEx610*[*papl-1::apl-1::gfp;punc-108::mCherry::unc-108*] | 20ng/μl, 5ng/μl |
