# Supplementary figures and images for: Intracellular Trafficking and Synaptic Function of APL-1 in Caenorhabditis elegans
Source: PLoS One. 2010 Sep 20;5(9):e12790. doi: 10.1371/journal.pone.0012790 (PMC2942829; doi:10.1371/journal.pone.0012790)

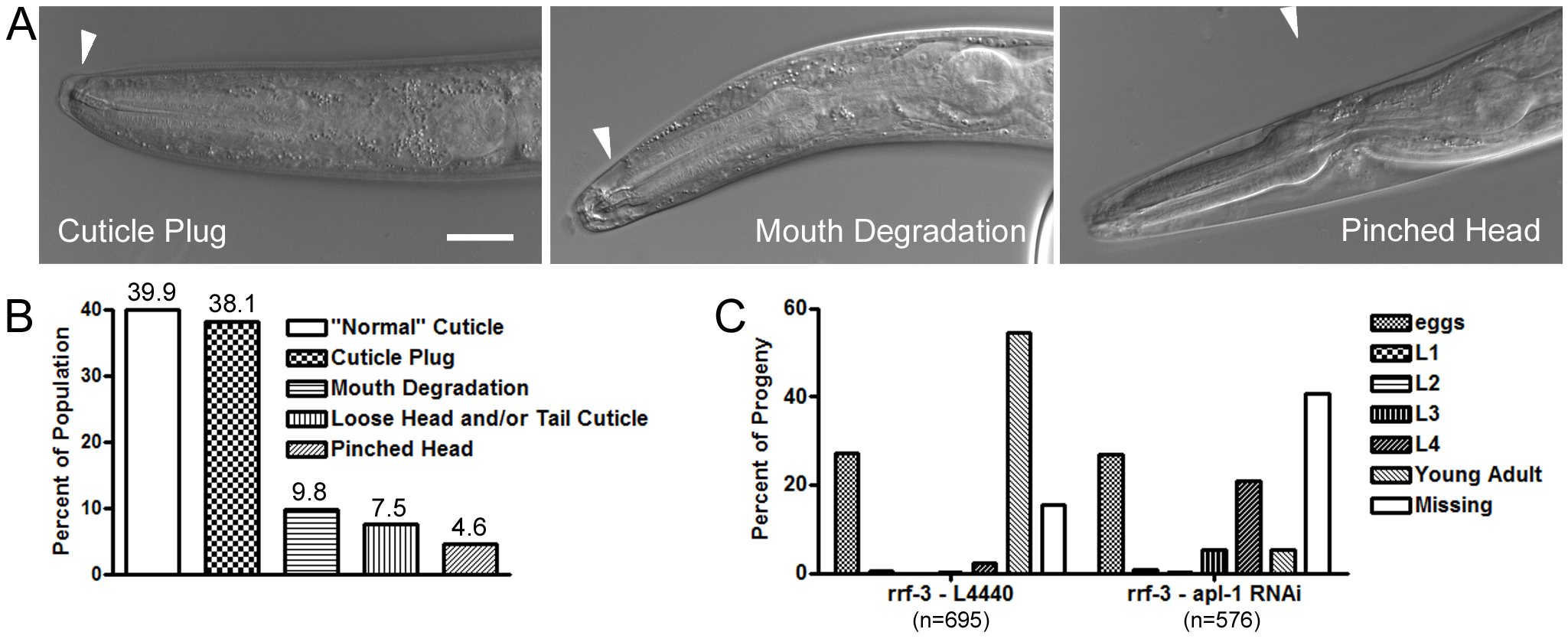

Supplement: Figure S1 — Molting defect seen during apl-1 RNAi. (A) Representative pictures of the various molting defects seen in RNAi sensitive strain rrf-3(pk1426) L4s during apl-1 knock-down. Arrowheads point to regions described by the inlaid text. (Scale bar, 20 µm.) (B) Percentages of the different molting defects seen in worms on apl-1 RNAi after 48 hours from egg stage (n = 173). (C) Percentage of different progeny stages 48 hours from egg stage on both control (L4440) and apl-1 RNAi. (1.06 MB TIF) [file pone.0012790.s002.tif]

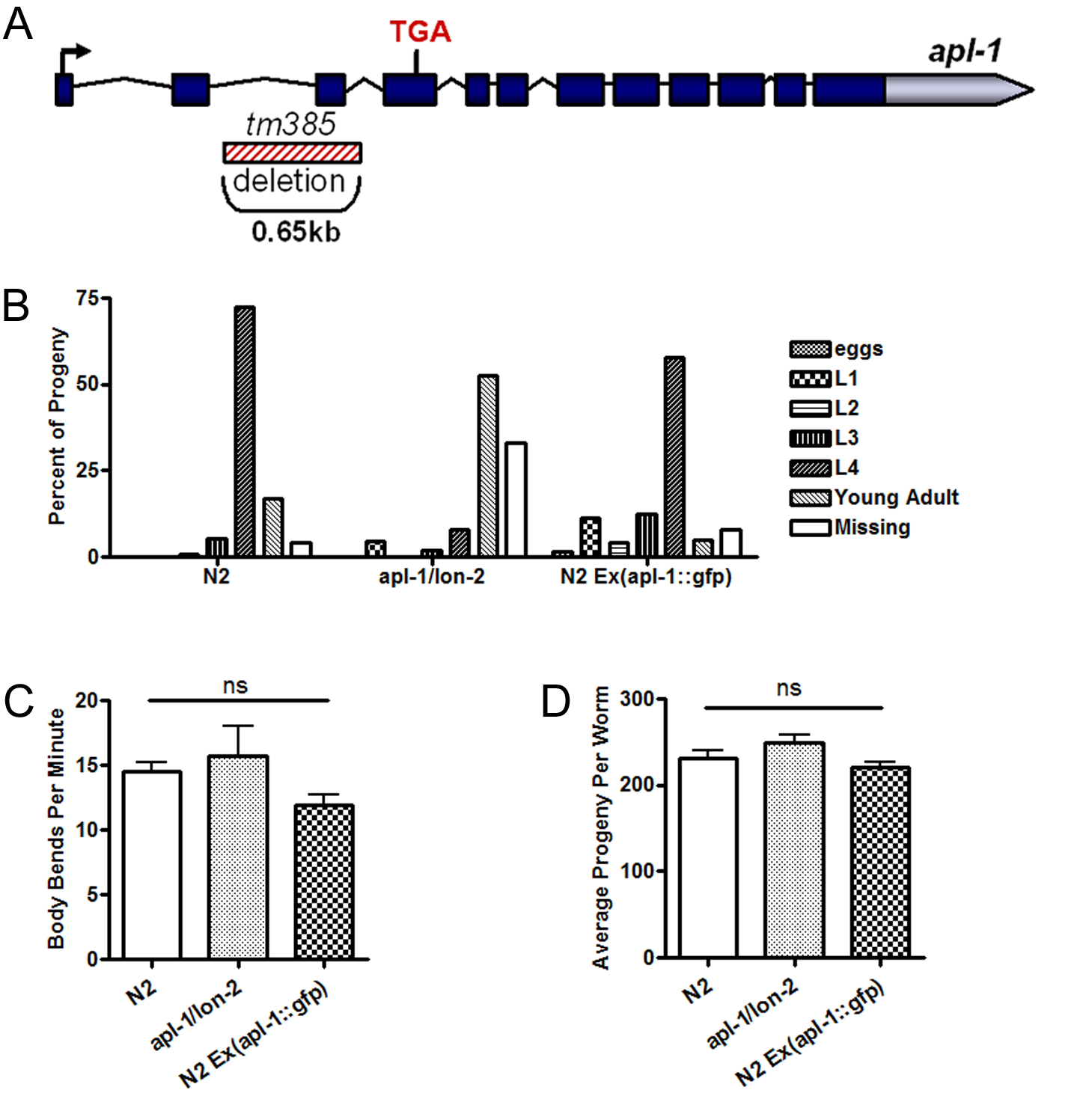

Supplement: Figure S2 — Rescue strains of apl-1 (tm385) have slower development. (A) Map of apl-1(tm385) deletion and premature stop codon. (B) Percentage of different larval stages after 48 hours of growth. The N2 strain and heterozygous apl-1(tm385)/lon-2(e678) are shown as negative controls. (N2, n = 697; apl-1/lon-2, n = 186; N2 Ex(apl-1::gfp)- 20 ng/µl, n = 528). Missing worms were those unaccounted for after 48 hours from the original total of eggs placed on the plate. These are likely in large proportion part of the L1 lethal population that are no longer visible. (C) No movement defects were detected in the transgenic APL-1::GFP strain. (N2, n = 14; apl-1/lon-2, n = 10; N2 Ex(apl-1::gfp)- 20 ng/µl, n = 14). (D) Brood sizes from the transgenic APL-1::GFP strain were normal when compared to the N2 and apl-1/lon-2 strains. (N2, n = 14; apl-1/lon-2, n = 11; N2 Ex(apl-1::gfp) - 20 ng/µl, n = 14). (*, P<0.05; **, P<0.01; ***, P<0.001.) Error bars represent the s.e.m. (0.64 MB TIF) [file pone.0012790.s003.tif]

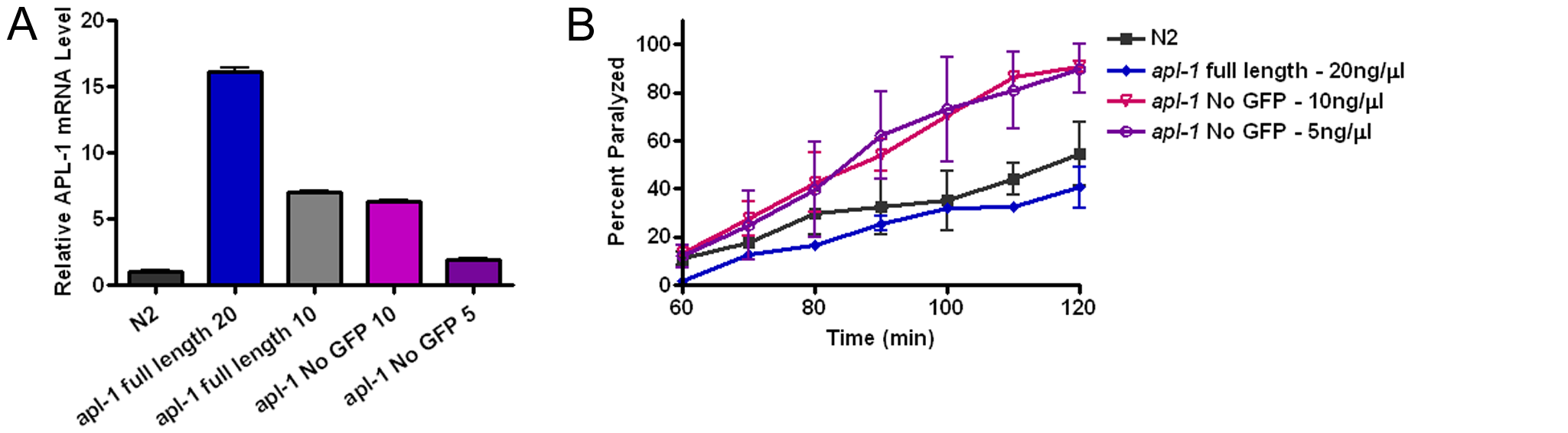

Supplement: Figure S3 — C-terminal GFP is not detrimental to the full length APL-1 rescue. (A) qRT-PCR of rescue strains showing comparative APL-1 expression with or without GFP. Error represents the STD. (B) Aldicarb experiment showing the retained hypersensitivity of full length APL-1 without the GFP tag at 10 ng/µl and 5 ng/µl. Error is the s.e.m. (0.28 MB TIF) [file pone.0012790.s004.tif]

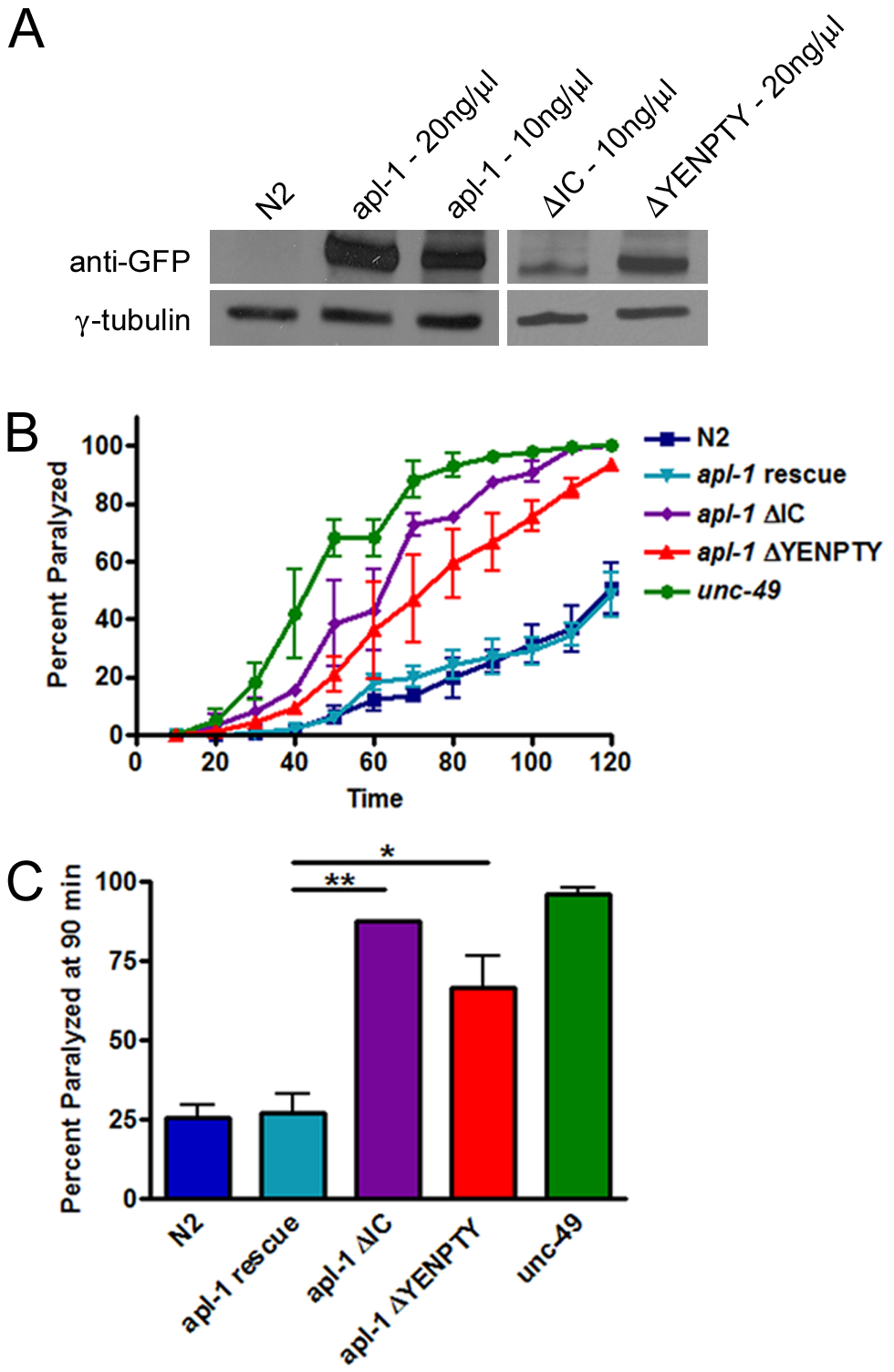

Supplement: Figure S4 — C-terminal mutation rescue strains cannot rescue the aldicarb hypersensitivity. (A) Western blot showing protein expression levels from the different rescue strains. (B) Rescue strains with either a C-terminal truncation or deletion of the YENPTY domain could not rescue the aldicarb hypersensitivity. (C) Quantification of the aldicarb experiment at the 90 min time-point. (*, P<0.05; **, P<0.01.) Error bars represent the s.e.m. (0.54 MB TIF) [file pone.0012790.s005.tif]

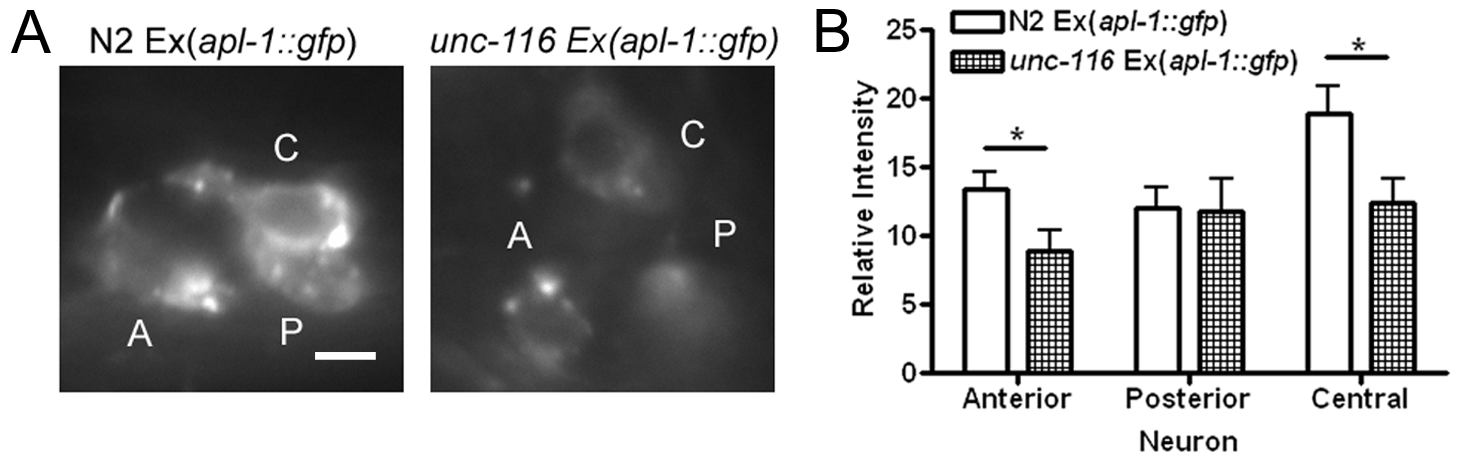

Supplement: Figure S5 — Loss of kinesin-1 function also leads to a reduction in APL-1::GFP fluorescence. (A) Representative pictures of head neurons with apl-1::gfp expression on N2 and unc-116 backgrounds. (Scale bar, 2.5 µm.) (B) Quantification of APL-1::GFP fluorescence (n = 10 per strain). (*, P<0.05.) Error bars represent the s.e.m. (0.41 MB TIF) [file pone.0012790.s006.tif]
